# Baroreflex and chemoreflex interaction in high-altitude exposure: possible role on exercise performance

**DOI:** 10.3389/fphys.2024.1422927

**Published:** 2024-06-04

**Authors:** Pablo Alvarez-Araos, Sergio Jiménez, Camila Salazar-Ardiles, Cristian Núñez-Espinosa, Valeria Paez, Maria Rodriguez-Fernandez, Antoine Raberin, Gregoire P. Millet, Rodrigo Iturriaga, David C. Andrade

**Affiliations:** ^1^ Exercise Applied Physiology Laboratory, Centro de Investigación en Fisiología y Medicina de Altura, Departamento Biomedico, Facultad de Ciencias de la Salud, Universidad de Antofagasta, Antofagasta, Chile; ^2^ Departamento de Kinesiología, Facultad de Ciencias de la Salud, Universidad de Atacama, Copiapó, Chile; ^3^ Escuela de Medicina de la Universidad de Magallanes, Punta Arenas, Chile; ^4^ Centro Asistencial de Docencia e Investigación (CADI-UMAG), Santiago, Chile; ^5^ Institute for Biological and Medical Engineering, Schools of Engineering, Medicine and Biological Sciences, Pontificia Universidad Católica de Chile, Santiago, Chile; ^6^ Institute of Sport Sciences, University of Lausanne, Lausanne, Switzerland; ^7^ Instituto de Ciencias Biomédicas, Facultad de Ciencias de la Salud, Universidad Autónoma de Chile, Santiago, Chile

**Keywords:** chemoreflex, carotid body, baroreflex, baroreceptors, high altitude exposure, breathing, ventilation, arterial pressure

## Abstract

The hypoxic chemoreflex and the arterial baroreflex are implicated in the ventilatory response to exercise. It is well known that long-term exercise training increases parasympathetic and decreases sympathetic tone, both processes influenced by the arterial baroreflex and hypoxic chemoreflex function. Hypobaric hypoxia (i.e., high altitude [HA]) markedly reduces exercise capacity associated with autonomic reflexes. Indeed, a reduced exercise capacity has been found, paralleled by a baroreflex-related parasympathetic withdrawal and a pronounced chemoreflex potentiation. Additionally, it is well known that the baroreflex and chemoreflex interact, and during activation by hypoxia, the chemoreflex is predominant over the baroreflex. Thus, the baroreflex function impairment may likely facilitate the exercise deterioration through the reduction of parasympathetic tone following acute HA exposure, secondary to the chemoreflex activation. Therefore, the main goal of this review is to describe the main physiological mechanisms controlling baro- and chemoreflex function and their role in exercise capacity during HA exposure.

## Introduction

High altitude (HA), characterized by reduced barometric and ambient O_2_ pressure, is challenging for human physiology (Huey, 2002; Windsor and Rodway, 2007; Mallet et al., 2021). Therefore, determining short- and long-term physiological adaptations to HA is fundamental. Chronic adaptations to HA involve maintaining oxygen (O_2_) supply to several tissues (Beall, 2007; Subudhi et al., 2014; Murray et al., 2018; Mallet et al., 2023). Altitude-born residents show physiological adaptations to cope with the hypoxia, such as a rise in minute ventilation at rest, high total lung capacity, lung diffusion, and better exercise performance than low-lander non-native residents (Greksa et al., 1988; Beall, 2007; Huerta-Sanchez et al., 2014; Simonson et al., 2015); however, not all high-altitude residents (i.e., Tibetans and Andeans) display the same adaptations (Beall, 2000; Beall, 2006; Bigham et al., 2013). Indeed, under similar hypobaric-hypoxic stress, Tibetans showed a one-half standard deviation higher resting ventilation and O_2_ saturation compared to Andeans; however, Andeans depict a higher hemoglobin concentration than Tibetans (Beall, 2006). Interestingly, Tibetans enhanced the hypoxic ventilatory response compared to the Andean population (Beall, 2000).

For non-residents, short-term acclimatization to HA includes an increment in arterial blood pressure, heart rate, metabolic demand, basal ventilation, autonomic alterations characterized by sympathoexcitation and parasympathetic withdrawal, and decreased exercise performance (Bartsch and Saltin, 2008; Naeije, 2010; Mallet et al., 2021). Recently, we found that the parasympathetic arm of the baroreflex (BR) pathway, measured through the phenylephrine-dependent increase of heart rate (HR), was reduced following acute exposure to HA (Beltran et al., 2020). In addition, a hypoxic-dependent potentiation of the chemoreflex was found, which modulates ventilation and autonomic functions in normoxia and during a hypoxic insult (Mahamed and Duffin, 2001). Therefore, it is likely that BR and chemoreflex pathways may interact at the central nervous system level (Somers et al., 1991; Katayama et al., 2019) during HA exposure. This idea is supported by the fact that peripheral carotid body (CB) denervation increases hypoxic-dependent BR range decrement in rats subjected to chronic intermittent hypoxia (Del Rio et al., 2015; Del Rio et al., 2016). In addition, (Kronsbein et al., 2020) also found that the buffering BR activity decreased during chemoreflex activation in normal human subjects (Kronsbein et al., 2020). Therefore, it is reasonable that the decreased BR-dependent parasympathetic activity found during acute exposure to HA (Beltran et al., 2020) results from a chemoreflex activation that may affect exercise performance during HA. (Machhada et al., 2017) found that optogenetic activation of the parasympathetic tone improved exercise capacity, producing a mimic of exercise training (Machhada et al., 2017). Additionally, they found that a decreased vagal control elicited using chemogenetics impairs exercise performance in rats (Machhada et al., 2017). It is also well-established that exposure to hypoxia alters sympathetic activation at rest (Perini et al., 1996; Sevre et al., 2001), which is related to decreased endurance performance (Schmitt et al., 2008). Hypoxia also alters the post-exercise parasympathetic reactivation, impairing recovery (Al Haddad et al., 2012; Fornasiero et al., 2018).

Exposure to HA negatively impacts alveolar pO_2_ and, consequently, arterial pO_2_, which has been linked to a marked decrease in exercise capacity (Naeije, 2010). The reduction in maximal O_2_ uptake (VO_2_max) is estimated at 6%–7% per 1,000 m increasing altitude (range 4.6%–7.5%) (Wehrlin and Hallen, 2006). However, an altered chemoreflex and BR function may contribute to decreased exercise performance, independent of environmental conditions (Pijacka et al., 2018; Eugenin et al., 2020). Therefore, considering the dependence of exercise performance on vagal activity (Machhada et al., 2017), HA-related chemoreflex activation (Iturriaga and Alcayaga, 2004), and decreased BR-dependent parasympathetic drive (Beltran et al., 2020), it is plausible to propose that the interaction between the chemoreflex and the BR may modulate the cardiorespiratory fitness through parasympathetic control, secondary to the decrease oxygen pressure due to HA. Thus, in the present review, we will analyze and discuss the role played by the chemoreflex and baroreflex and their effects on physical capacity during HA exposure.

### Baroreceptors and the arterial baroreflex control

The BR is a homeostatic mechanism that maintains the cardiac output at normal levels through negative feedback in the brainstem, modulating heart rate and arterial blood pressure by controlling sympathetic and parasympathetic activities (Thrasher, 2002). Thus, an increment in arterial blood pressure produces stimulation of the baroreceptors located in the aortic arch and carotid bifurcation, increasing parasympathetic cardiac response and attenuating sympathetic outflow to the heart and peripheral vessels, triggering a decrease in HR, cardiac contractility, and peripheral resistance. Conversely, decreased arterial blood pressure reduces the neural discharges of the baroreceptors, leading to increased sympathetic drive, vasoconstriction, and hypertension, producing a positive chronotropic response and increased cardiac contractility. Mechanistically, the BR is initiated in the baroreceptors, which are mechanically sensitive nerve endings and found in the aortic arch and the carotid bifurcation (Persson et al., 1988). The aortic baroreceptor afferent nerve fibers are conveyed in the aortic depressor nerve, with their soma located in the nodose ganglion (NG).

On the other hand, the carotid baroreceptors are found in the carotid sinus, with the soma lying in the petrosal ganglion (PG) (Fadel et al., 2003; Kougias et al., 2010; Lau et al., 2016). The arterial pressure stretches the carotid sinus of the aortic arch, inducing a rise of cytosolic Ca^2+^. Nevertheless, the cytosolic Ca^2+^ was higher in aortic baroreceptor neurons than in carotid baroreceptor neurons, suggesting aortic baroreceptors are more sensitive to arterial blood pressure changes than carotid baroreceptors (Lau et al., 2016). Indeed, it has been shown that, through baroreceptor nerve activity *in vivo*, aortic depressor nerve discharge was increased compared to the carotid sinus nerve activity (afferent activity), a similar change in blood pressure in rats (Lau et al., 2016).

#### Baroreflex-dependent sympathetic and parasympathetic intracellular mechanisms of heart rate control

*Cardiac sympathetic mechanism.* The baroreflex-dependent sympathetic activation (HR increment) is mediated by norepinephrine (NE) releases, which bind to the β-adrenergic receptors activating Na^+^ channels (Kaupp and Seifert, 2001; Lakatta and DiFrancesco, 2009). In addition, the β-adrenergic receptor controls intracellular Ca^2+^ control (Fadel et al., 2003) through cyclic adenosine 3′,5′-monophosphate (cAMP)/cAMP-dependent protein kinase (PKA) signaling (Gray et al., 1998). PKA phosphorylation is mediated by the A-kinase-anchoring protein (AKAP-15/18), which interacts with the intracellular domain of the channel and brings the PKA to its binding site (Sampson and Kass, 2010). A similar process occurs in the sarcoplasmic reticulum, where AKAP-6 interacts with the ryanodine channels and recruits the PKA site, increasing the release of intracellular [Ca^2+^], which contributes to increased HR and contractility rate (Lakatta and DiFrancesco, 2009). For instance, in the NE-mediated chronotropic response, there must be a fast removal of [Ca^2+^]i, which is performed by mitigating the inhibition of the Ca^2+^ATPasa pump by phosphorylation of the phospholamban protein (Marx et al., 2002).

*Cardiac parasympathetic mechanism.* The HR reduction is mediated by muscarinic receptors (M2), acetylcholine-dependent receptors (Kaupp and Seifert, 2001; Thrasher, 2002). Muscarinic receptors are expressed in the sinoatrial, atrioventricular, and cardiomyocyte T-tubules system (Kaupp and Seifert, 2001). The activation of the M2 receptor is mediated by a G protein-coupled receptor, which rectifies K^+^ conductance and decreases cAMP, reducing PKA activation (Swynghedauw, 1999). All these produce a longer duration of atrial action potentials and consequently decrease the HR and contractility rate of the heart (Olshansky et al., 2008). In addition, M2 receptors activate nitric oxide synthase (NOS) via guanylate cyclase, inhibiting L-type Ca^2+^ channels (Olshansky et al., 2008) and slowing the entry of Ca^2+^ into the intracellular medium, which contributes to decreasing the contractility rate of the heart (Swynghedauw, 1999; Olshansky et al., 2008).

Previously, we found a baroreflex-mediated parasympathetic withdrawal during HA exposure. Indeed, we observed a diminished bradycardic response to phenylephrine and a decreased power spectral density at a high-frequency component (parasympathetic drive) of the time-varying heart rate variability, evidencing a marked decrease in vagal outflow (Beltran et al., 2020). The autonomic control of physical performance is of such relevance that decreased parasympathetic drive triggers performance impairment, while increased parasympathetic control promotes a mimicry of exercise training, improving physical performance in rats (Machhada et al., 2017).

### Carotid body chemoreceptor and chemoreflex function

The carotid body (CB) is the main peripheral oxygen chemoreceptor, composed of chemoreceptors (glomus or type I cells) and sustentacular clusters type II cells (Iturriaga and Alcayaga, 2004; Prabhakar, 2006; Iturriaga et al., 2021b). The CB type I cells respond to a wide variety of stimuli, such as changes in arterial levels of pO_2_, carbon dioxide pressure (pCO_2_), pH, blood flow, glucose, temperature, osmolarity, and insulin; therefore, they are considered polymodal receptors (Gonzalez et al., 1994; Ding et al., 2011; Iturriaga et al., 2021b). In response to low pO_2_ and high pCO_2_-H^+^, type I cells are activated by inhibiting O_2_-sensitive K^+^ channels. Further, the hypoxia-dependent production of gasotransmitters (NO, CO, H_2_S) also regulates ion channel activity in the CB (Iturriaga et al., 2021a). The intracellular pathways related to the neurotransmitter release are AMP-activated protein kinases and PKC, as well as reactive oxygen species in the CB type I cells, promoting the release of neurotransmitters such as acetylcholine (Ach) and adenosine triphosphate (ATP) that interact with receptors in the nerve terminal of petrosal sensory neurons that project through the carotid sinus nerve to the nucleus tractus solitarii (NTS) (Iturriaga and Alcayaga, 2004). In addition, the type I cells also release several molecules, which serve as excitatory or inhibitory modulators of CB chemosensory transduction (i.e., NO, histamine, and Ang II) (Iturriaga and Alcayaga, 2004; Del Rio et al., 2008).

Briefly, a reduction in pO_2_ in the arterial blood is detected by primary O_2_ sensors, type I carotid body cells, which rapidly communicate with potassium (K^+^) channels, leading to the closure of these channels. In turn, via membrane depolarization and increases in intracellular [Ca^2+^] concentration, the release of neurotransmitters (i.e., Ach and ATP) leads to excitation of the afferent nerve that runs in the carotid nerve sinus up to the respiratory centers in the brain stem (Teppema and Dahan, 2010).

Additionally, it has been evidenced that the CBs are related to exercise capacity in physiological and pathophysiological conditions (Honda et al., 1979; Honda, 1985; Andrade et al., 2021a). CB denervation or resection and exposure to 100% O_2_ (resulting in decreased CB activity) reduce ventilatory responses in exercise and markedly diminish exercise capacity in humans and animals. Indeed, hyperoxic gas applied during ventilatory threshold 2 (VT2) decreases pulmonary ventilation in humans, suggesting that the CB contributes at least in part to increasing ventilation at VT2 during incremental exercise (Masuda et al., 1988). Further, Honda et al. (1979) showed that CB resection in asthma patients decreases the respiratory response to exercise compared to patients with intact CBs (Honda et al., 1979). Along with this, we showed that CB resection promotes a phenotype shift from heart failure tolerant to physical exercise animals to intolerants (Andrade et al., 2021b). All this evidence strongly suggests the pivotal role of CB peripheral chemoreceptors in ventilatory response to physical exercise as well as training-dependent adaptations.

**FIGURE 1:**
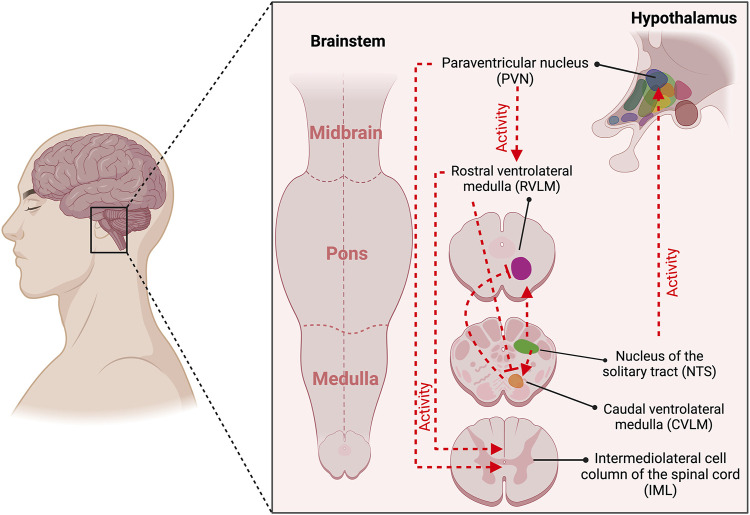
Central command is associated with the activation of baroreceptors and chemoreceptors. The first central integration of sensory information from peripheral chemoreceptor and baroreceptor inputs occurs in the commissural and medial divisions of the nucleus of the solitary tract (NTS). The NTS neurons integrate and project to other cardiac autonomic and respiratory regions (i.e., rostral ventrolateral medulla [RVLM], caudal ventrolateral medulla [CVLM], and the paraventricular nucleus (PVN). Baroreceptor stimulation promotes increased synaptic activity from NTS to CVLM, which projects a gamma-aminobutyric acid (GABA)-mediated activity to RVLM, reducing sympathetic drive. Chemoreceptor activation increases synaptic transmission from NTS-RVLM-IML, raising sympathetic drive. Besides, NTS-mediated chemoreceptor activation increases their activity to PVN and subsequently to RVLM and IML, promoting sympathetic activation. Created with BioRender.com.

Figure 1 depicts the neural control of chemoreflex function. During hypobaric hypoxic environments, chemoreceptor activation promotes ventilatory acclimatization and sympathoexcitation (Vizek et al., 1987; Schultz and Sun, 2000). The first central integration of sensory information from peripheral chemoreceptor and baroreceptor inputs occurs in the commissural and medial divisions of the nucleus of the solitary tract (cNTS and mNTS, respectively) (Claps and Torrealba, 1988; Finley and Katz, 1992). The cNTS and mNTS neurons integrate and project to other autonomic and respiratory regions (i.e., rostral ventrolateral medulla [RVLM], caudal ventrolateral medulla [CVLM], and the central pattern generator [CPG]) (Ponikowski et al., 2001; Rosin et al., 2006; Smith et al., 2010; Diaz et al., 2020). King et al. (2012) showed that acute-hypoxic stimulus increments the activation of cNTS catecholaminergic neurons (King et al., 2012). In addition, it has been found that sustained hypoxia, similar to HA exposure, enhances NTS glutamatergic synaptic transmission after 1 day and augments glutamate (Glu) receptor expression after 7 days (Zhang et al., 2009; Pamenter et al., 2014; Accorsi-Mendonça et al., 2015; Accorsi-Mendonça et al., 2019). Other regions sensitive to hypoxia are RVLM and CVLM (King et al., 2013; Boychuk et al., 2012; D'Agostino et al., 2001); nevertheless, it has been demonstrated that the activation of RVLM is CB-dependent and not a direct hypoxic effect (Del Rio et al., 2013). Interestingly, we found BR-dependent autonomic control impairment during HA exposure (3,290 m) (Beltran et al., 2020); however, whether it depends on the chemoreflex activation with their respective neural autonomic nuclei activation has not been demonstrated. Additionally, although considering that autonomic control and chemoreceptors are critically essential to maintaining cardiorespiratory fitness during exercise, there is no comprehensive evidence depicting whether, during HA or normobaric hypoxia, the exercise capacity impairment is related to BR-dependent parasympathetic withdrawal, secondary to a chemoreflex enhancement.

### Effects of hypoxia on the baroreflex (BR)

The cardiovagal baroreflex is challenged in numerous conditions, such as during altitude exposure (Bourdillon et al., 2018a; Hermand et al., 2021; Bourdillon et al., 2023). However, understanding the underlying mechanisms of the effect of HA on BR remains limited (Olshansky et al., 2008; Beltran et al., 2020). Interestingly, chronic intermittent hypoxia (Swynghedauw, 1999; Freet et al., 2013), high altitude (Bourdillon et al., 2017a; Bourdillon et al., 2018a; Beltran et al., 2020; Bourdillon et al., 2023), and suffocation (Gu et al., 2007) promote similar physiological responses characterized by modified autonomic control assessed through heart rate variability (HRV) (sympathoexcitation and parasympathetic withdrawal) and, moreover, a marked decrease of BR assessed through sequence methods. Therefore, it is possible to suggest that the evidence at high altitude and chronic intermittent hypoxia could be, in part, comparable. Indeed, of interest is that the influence of barometric pressure seems negligible since no differences in the decreased baroreflex sensitivity were found between normobaric hypoxia and hypobaric hypoxia (Bourdillon et al., 2017b). It has been shown that after 30 days of chronic intermittent hypoxia exposure, which promotes hypertension (from the second day of hypoxia), there is a decrease in BR function and an increase in sympathetic outflow in conscious rats (Lai et al., 2006; Zoccal et al., 2009; Freet et al., 2013; Del Rio et al., 2016). However, the evidence is controversial, and no changes in BR-dependent splanchnic nerve activity after chronic intermittent hypoxia have been found (Prabhakar et al., 2012). Similarly, rats exposed to 7 days of intermittent hypoxia do not show any effects on BR function (Faulhaber et al., 2012). Moreover, the controversy is such that Zoccal et al. (2009), using heart-brain stem preparation, reported increased BR function in juvenile rats subjected to chronic intermittent hypoxia (Zoccal et al., 2009). Independent of the controversy, it has been proposed that hypoxic-dependent BR depression could be due to a reduction in the carotid baroreceptor activity rather than a direct effect on brainstem autonomic nuclei (Lesske et al., 1997). Nevertheless, rats exposed to chronic intermittent hypoxia improved BR sensitivity after CB denervation, even though the animals were still hypoxic, suggesting that the chemoreflex could be hierarchically superior to the BR (Del Rio et al., 2016).

### Effect of hypoxia on hypoxic chemoreflex

**FIGURE 2:**
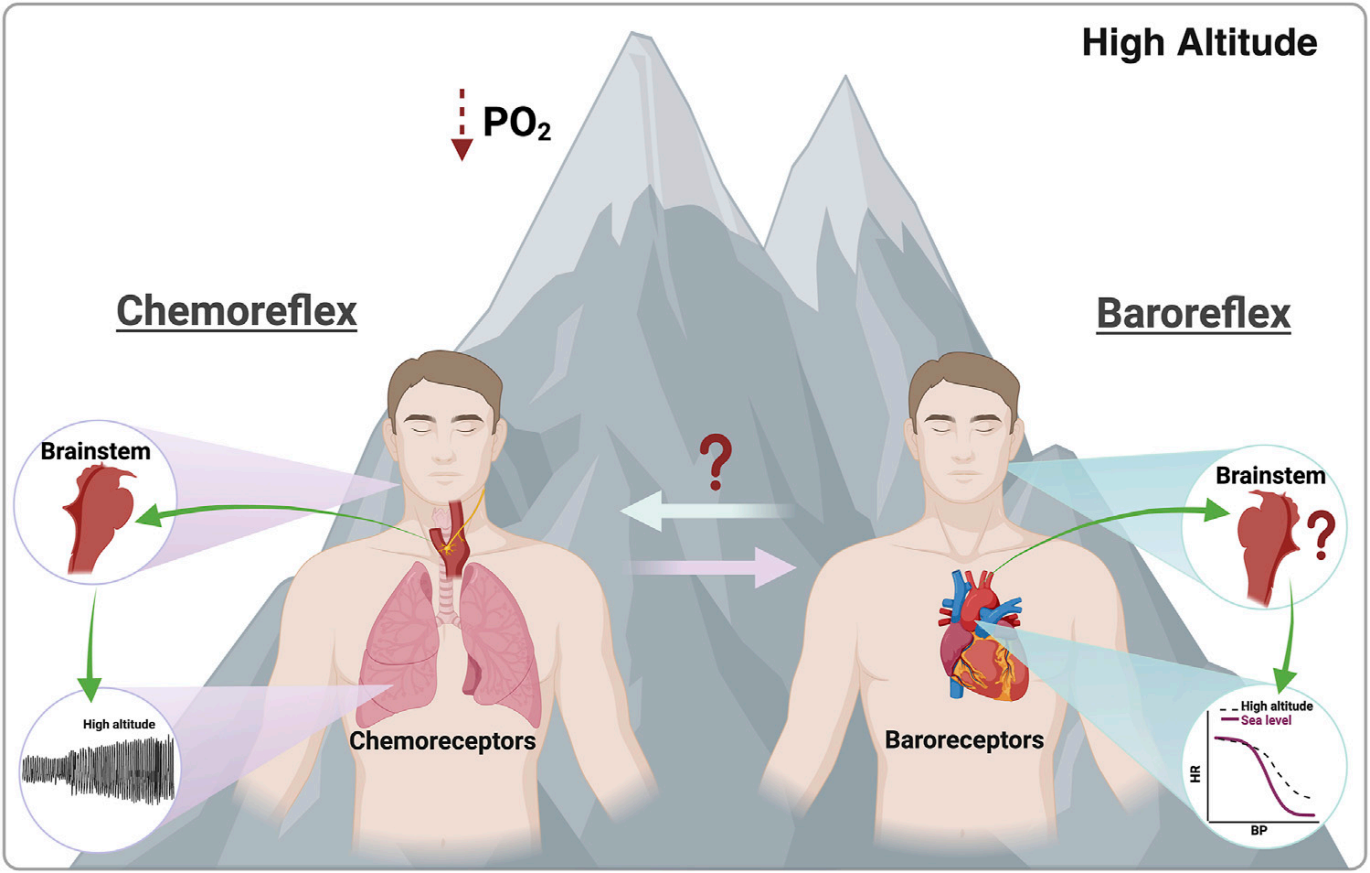
Effects promoted by chemoreflex and baroreflex activation. During high-altitude exposure, peripheral chemoreceptors are activated due to a PO2 reduction. Contrarily, during this environmental insult, the parasympathetic-dependent baroreflex control is reduced due to an overall autonomic control impairment. Notably, there is no evidence of the effect of high-altitude exposure on baroreflex-dependent central command and baroreceptors or the possible interaction between baroreflex and chemoreflex function. Created with BioRender.com.

During HA exposure, the human body responds at several levels, from cellular to whole-body, encompassing early and late responses. Early responses to HA involve ventilatory and circulatory adjustments, increased basal ventilation, autonomic imbalance marked by elevated release of epinephrine and norepinephrine, changes in acid-base balance, decreased glomerular filtration rate, and impaired pO_2_ and pCO_2_ exchange, and as a consequence a reduced exercise performance (Beall, 2007; Naeije, 2010; Farias et al., 2013; Huerta-Sanchez et al., 2014; Simonson et al., 2015; Mallet et al., 2021; Mallet et al., 2023). Late responses are characterized by gene overexpression, including hypoxic inducible factor [HIF]1α, HIF2α, vascular endothelial growth factor [VEGF], and VEGF receptor. Additionally, there is an increase in erythropoietin and hemoglobin, which are concomitant with an increase in hematocrit, leading to elevated blood viscosity. Other late responses include the activation of the renin–angiotensin–aldosterone system and alterations in intra- and extra-vascular fluid distribution (Beall, 2007; Naeije, 2010; Farias et al., 2013; Huerta-Sanchez et al., 2014; Simonson et al., 2015). Interestingly, most early responses are CB-mediated (Iturriaga et al., 2021b; Arce-Álvarez et al., 2022) (Figure 2). Regarding CB-mediated hypoxic ventilatory response, this exhibits several phases: i) initial short-term hyperpnea (Eldridge, 1974; Badr et al., 1992); ii) with continued hypoxic stimulus, there is time-dependent hyperventilation and sympathoexcitation that may persist for several hours or days (Wang et al., 2008); iii) if hyperventilation and increased sympathetic drive persist, it is possible to observe the decline of ventilation and not sympathoexcitation after several days of hypoxia (Hansen and Sander, 2003; Dempsey et al., 2014). Although peripheral chemoreceptor stimulation affects several organs in the body, most changes over the first hours of permanence in hypoxia occur at the level of the respiratory and cardiovascular systems (Bartsch and Gibbs, 2007). Notably, hypoxic-dependent local vasodilation is inhibited by CB (sympathetic)-mediated vasoconstriction in more metabolically activated organs (i.e., skeletal muscles during exercise) (Kumar and Bin-Jaliah, 2007). Vascular hyperactivity promotes two main effects: increment of arterial blood pressure and redistribution of blood flow, both relevant phenomena to physical fitness (Heistad and Abboud, 1980; Kumar and Bin-Jaliah, 2007; Dempsey and Smith, 2014). Accordingly, as was mentioned, chemoreflex activation promotes several physiological changes, which have been associated with homeostatic functions to meet metabolic demand. Then, considering that physical exercise triggers a greater metabolic requirement, it is possible to propose that CB may partially elicit cardiorespiratory and vascular responses by altering the BR function during HA exposure (Figure 2).

### Reciprocal effect of physical exercise and baroreflex

The BR regulates hemodynamics during exercise (Fukuma et al., 2012; Dipla et al., 2013), which is dependent on age (Grassi et al., 2004; Fukuma et al., 2012), sex (Fukuma et al., 2012), and body fat distribution (Laterza et al., 2007). Further, decreased BR sensitivity has been associated with increased cardiovascular risk, cardiac electrical instability, and orthostatic intolerance (Fukuma et al., 2012). Otherwise, a normal BR function would ensure an appropriate cardiovascular response during exercise, regulating cardiac output (Fukuma et al., 2012). Conversely, an inappropriate high dose of exercise was associated with decreased baroreflex sensitivity, which may be used to diagnose overtraining (Bourdillon et al., 2018b). In physiological conditions, exercise training, similar to hypoxia, can promote resetting the BR function (Halliwill et al., 2003), shifting the BR to operate at higher blood pressure. In contrast, exercise training can induce robust effects on BR control in pathophysiological states, increasing the range, slope, and gain. However, only 25% of hypertensive subjects under chronic aerobic training could stabilize blood pressure, which could be associated with chronic morphological changes and not necessarily BR sensitivity (Liu et al., 2012). Indeed, it has been shown that exercise training can reduce sympathetic activity and increase BR-dependent parasympathetic function, determined through the sequence method, in an animal model of heart failure (Liu et al., 2000; Andrade et al., 2017). In addition, improvement in BR gain after an aerobic training program has been reported in pre-hypertensive and hypertensive subjects (Bertagnolli et al., 2008; Fisher et al., 2012; Liu et al., 2012). The effects of exercise training on BR are limited to cardiovascular diseases, with no demonstrated impact on metabolic disorders, such as diabetes mellitus. Indeed, Dipla et al. (2013) showed no significant association between changes in BR sensitivity in patients suffering from diabetes mellitus after applying a training program (Dipla et al., 2013). The evidence is controversial, and no effect of exercise training on BR has been found. Indeed, carotid BR sensitivity has remained unchanged after an aerobic training program compared with control subjects (Goldberg et al., 2012). Despite the controversy, the prevailing evidence indicates that exercise training significantly improves BR gain and triggers a resetting of the BR control. Nevertheless, it remains unclear whether the deterioration of cardiac BR gain during HA (Beltran et al., 2020) can be improved or remains unchanged compared to sea level following an exercise training intervention. Accordingly, further research is needed to address whether exercise training could be a feasible strategy to counteract the reduction of HA-induced BR gain.

### Reciprocal relationship between exercise and hypoxic chemoreflex

CB is essential to maintain eupneic ventilation (Olson et al., 1988). However, although the evidence suggests that CB could be pivotal in cardiorespiratory response to exercise, apparently, the exercise did not modify CB chemoreflex, except in pathophysiological conditions (Andrade et al., 2018a; Wan et al., 2023). Thus, when CB-dependent hyperreflexia in autonomic-related diseases (i.e., heart failure, hypertension, etc.) is observed, exercise training reduces CB-dependent sympathetic overdrive and hypoxic ventilatory response (Schultz and Sun, 2000; Schultz et al., 2015). One of the first observations showing the relevance of CB chemoreceptors during exercise was made by Weil et al. (1972). They found that the hypoxic breathing response increased during moderate exercise compared to a resting condition, suggesting a potential association with the cardiorespiratory response to physical effort (Weil et al., 1972). Interestingly, peripheral chemoreceptor activation, similar to physical exercise, induces sympathoexcitation, promoting vasoconstriction at several levels, such as skeletal muscles and renal and mesenteric vascular beds (Seals et al., 1991; Gonzalez et al., 1994; Buckwalter and Clifford, 1999). Although vasoconstriction itself may limit blood flow to the muscle during exercise (Joyner et al., 1992), it has been proposed that exercise-dependent sympathoexcitation helps to distribute the blood flow to the active muscles according to their metabolic demand (Buckwalter and Clifford, 1999; Stickland et al., 2007). Even though sympathoexcitation during exercise is well-established, no conclusive evidence defines the principal sensor during exercise. It has been proposed that metaboreflex, exercise pressor reflex (constitute of metabo- and mechano-reflex), baroreflex, and chemoreflex are involved in cardiopulmonary response to exercise (Wan et al., 2023). Indeed, dopamine-dependent inhibition of chemoreflex decreases α-adrenoreceptor-mediated vasoconstriction, which augments blood flow to human active muscles (Stickland et al., 2011). Another mechanism that could increase ventilation during exercise is the increase in temperature. Nevertheless, CB is not essential in hyperventilation associated with exercise-induced hyperthermia (Daniłowicz-Szymanowicz et al., 2010; Fujii et al., 2019). Fujii et al. (2019) showed that the decrease in temperature and exposure to 100% O_2_ does not differentiate the ventilatory response to an incremental exercise, suggesting that peripheral chemoreceptors are irrelevant to exercise-induced hyperthermia in humans. The evidence indicates that the peripheral chemoreflex is involved in the ventilatory response to exercise. Nevertheless, there is no evidence whether the cardiorespiratory fitness deterioration during hypobaric hypoxia is due to altered chemoreflex function and whether it could be through an interaction with the BR.

### Cardiorespiratory and metabolic response to exercise and during HA exposure: implication for chemoreflex and baroreflex control

It is well known that during physical activity, there is an increase in energy demand and, consequently, in cardiopulmonary and O_2_ uptake to meet the energy requirements (Hill and Lupton, 1923). In normoxia, during the initial phase of an incremental exercise, there is a cardiodynamic response, principally characterized by an increase in HR and stroke volume, with little contribution from ventilation to O_2_ consumption (Housh et al., 1991; McLellan and Cheung, 1992; Pringle and Jones, 2002; Whipp et al., 2005). It has been proposed that this cardiodynamic response is “associated” with the exercise pressor reflex (type III and IV afferent feedback fibers in muscles) and not with BR and chemoreflex, triggering an increase in ionotropic and chronotropic heart responses mediated by the activation of the sympathetic system (McCloskey and Mitchell, 1972; Amann et al., 2010; Tocco et al., 2015). At ventilatory threshold 1, there is a significant increase in pulmonary ventilation, which occurs in tandem with an increase in HR and lactate (Hofmann and Tschakert, 2017). Then, at ventilatory threshold 2, there is an abrupt increase in pulmonary ventilation and a significant increment of systemic lactate concentration (>4 mM) (Hofmann and Tschakert, 2017). It is currently under discussion whether chemoreceptors can detect lactate; therefore, the ventilatory response during exercise could be related to CB activation (Chang et al., 2015; Torres-Torrelo et al., 2021). Nevertheless, it has been shown that CB chemoreceptor cells do not respond to lactate in Wistar Kyoto rats (Spiller et al., 2021). In summary, during an incremental exercise, there is a complex, highly coordinated physiological mechanism encompassing pulmonary, cardiovascular, metabolic, and autonomic responses that enable the proper delivery of O_2_ to the active tissues. However, whether these mechanisms are modified during short-term HA exposure, which may be associated with chemo-baroreflex interaction, is not known yet.

**FIGURE 3:**
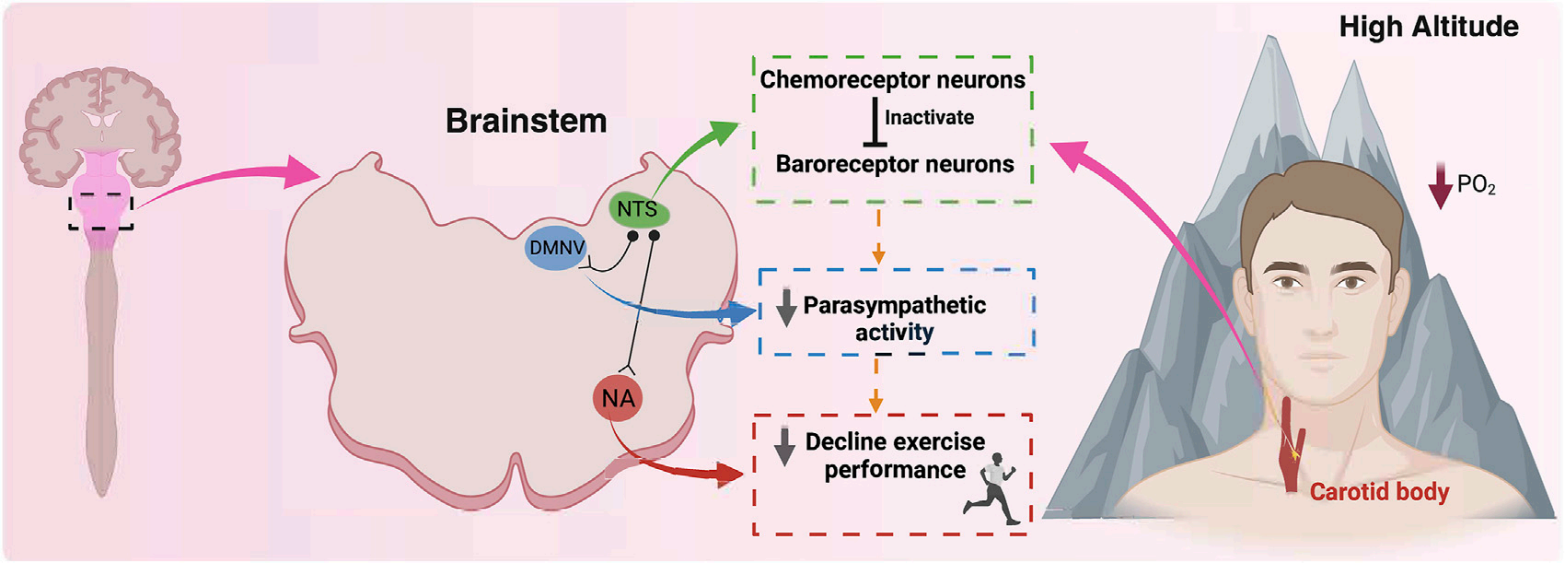
A hypothetical proposal related to the role of chemoreceptor and baroreceptor interaction and their effects on exercise performance at high altitudes. During high altitude exposure, there is an activation of the carotid body (CB) peripheral chemoreceptors, which at the brainstem level, specifically in the nucleus of the tractus solitarii (NTS) activate chemoreceptor neurons. At NTS, chemoreceptor neurons inhibit baroreceptor neurons, reducing their activity to parasympathetic neurons, such as the nucleus ambiguous (NA) and dorsal motor nucleus of the vagus (DMNV), consequently reducing baroreflex control and parasympathetic drive. Finally, the reduction of the vagal control negatively impacts exercise performance at high altitudes. Therefore, we propose that chemoreceptor neurons inactivate baroreceptor neurons, reducing parasympathetic drive and contributing to a decline in exercise performance at high altitudes, secondary to a reduction in barometric pressure. Created with BioRender.com.

As mentioned before, under resting conditions, HA promotes an increase in minute ventilation and enhances cardiac output, ensuring an adequate oxygen supply to the tissues (Klausen et al., 1966; Naeije et al., 1982; Cremona et al., 2002). It has been shown that a decrease of 12% in the inspired fraction of O_2_ can promote an increase of ∼22% in cardiac output, which was explained mainly by the increase of HR (∼18%) in healthy subjects (Naeije et al., 1982), decreasing the HR reserve. In addition, concomitant to cardiopulmonary adjustment, there is a marked autonomic control impairment characterized by a BR-dependent parasympathetic withdrawal during HA exposure (Naeije et al., 1982; Beltran et al., 2020). Regarding exercise at HA, it is well-established that the VO_2_max, exercise performance, and functional capacity are markedly reduced during HA exposure (Dempsey et al., 1972; Maher et al., 1974; Young et al., 1996; Fulco et al., 1998; Bassett and Howley, 2000; Millet et al., 2010; Andrade et al., 2018b; Burtscher et al., 2018). Indeed, highly trained running athletes evidenced a small but significant aerobic performance deterioration, even at 540 m (Fulco et al., 1998) or 580 m (Gore et al., 1996). Alexander et al. (1983) also found evidence that the VO_2_max is affected by HA, observing a decrease of 25% in maximum aerobic capacity at an altitude of 3,100 m (Alexander and Grover, 1983). Similarly, we found that aerobic time-trial performance decreased by ∼25% during acute exposure to 3,350 m in healthy individuals (Andrade et al., 2018b). Moreover, VO_2_max is expected to decrease by 0.9% per every 100 m over 1,100 m above sea level (Vogt and Hoppeler, 2010). All these phenomena have been attributed directly to the decrease in partial pressure and arterial SpO_2_ resulting from lower barometric pressure at HA, affecting the oxygen supply to active organs (Wagner, 2010). Slight differences have been reported between normobaric and hypobaric hypoxia (Millet and Debevec, 2020). However, the resting and exercise adjustments at HA suggest that autonomic control (chemoreflex and baroreflex) could play an important role in exercise performance during short-term HA exposure. Nevertheless, it is worth mentioning that there is no evidence indicating a chemo-baro interaction during HA exposure that could explain the reduction in exercise performance independent of reduced partial O_2_ pressure (Figure 3).

## Future directions

Chemoreflex and baroreflex responses and interaction are modified during HA exposure, leading to a marked deterioration in exercise performance. Additionally, it has been determined that parasympathetic control is a determinant of exercise capacity. Therefore, it is reasonable to suggest that further research should focus on the role of chemoreflex and baroreflex-dependent vagal deterioration in the impairment of physical effort during HA exposure. It would be necessary to determine if the brainstem nuclei interact with the BR and the chemoreflex pathways, and their physiological consequences on exercise performance during hypobaric hypoxia. Finally, considering the critical importance of physical capacity at HA in several human activities (i.e., tourism, sports, border security, and mining) and the functional capacity in several chemoreflex/baroreflex-altered pathophysiological states, further research should not only elucidate the roles of chemoreflex and baroreflex separately but also explore potential pharmacological and non-pharmacological strategies to modify them as critical nodal points. This research should be broad in scope, focusing on physical performance and daily activities at high altitudes.

## Conclusion

Hypoxic environments, while capable of harboring life, are inhospitable places where organisms struggle to survive. HA houses several human activities, such as tourism, sports, border security, and mining, which justify the research from basic to clinical science. Chemoreceptors and baroreceptors pathways share some brainstem neural nuclei, which are activated during hypoxia, making them susceptible to intervention and improving human whole-body response to hypoxia. Indeed, the present review focuses on chemoreflex and baroreflex interaction and their possible role in exercise capacity during HA exposure. The evidence suggests that an alteration of the chemoreflex could precede baroreflex-dependent parasympathetic withdrawal, resulting in a secondary impact on exercise performance at HA; however, this hypothesis has not yet been elucidated. Thus, our manuscript summarizes the literature and proposes new hypotheses that need to be addressed in the future.
